# Impact of COVID‐19 on digital practice in UK paediatric speech and language therapy and implications for the future: A national survey

**DOI:** 10.1111/1460-6984.12750

**Published:** 2022-08-04

**Authors:** Rafiah Patel, Elena Loraine, Mélanie Gréaux

**Affiliations:** ^1^ City, University of London and St George's University Hospitals NHS Foundation Trust London UK; ^2^ Central London Community Healthcare London UK; ^3^ University of Cambridge Cambridge UK

**Keywords:** COVID‐19, paediatric speech and language therapy, survey, telehealth, telepractice, UK

## Abstract

**Background:**

The COVID‐19 pandemic and subsequent measures to reduce transmission risk has led to unprecedented digital transformation across health, education and social care services. This includes UK paediatric speech and language therapy (SLT), which sits at the crossroads of these services. Given the rapid onset of this pandemic‐induced digital transition, there is now a need to capture, reflect and learn from the SLT profession so that benefits can be sustained, and barriers addressed.

**Aims:**

To survey the impact of COVID‐19 remote working on UK paediatric SLTs’ digital views and experiences using the Capability, Opportunity, Motivation and Behaviour (COM‐B) model.

**Methods & Procedures:**

An online survey was conducted from May to October 2020. Respondents were asked to rate their use of technology in service delivery before and during the pandemic, to select factors facilitating digital practice, and to provide open‐response aspirations for the future role of technology in paediatric SLT which were analysed thematically using the COM‐B behaviour change model.

**Outcomes & Results:**

A total of 424 UK paediatric SLTs responded to the survey. Findings indicate a marked increase in clinicians’ perception of their frequency, convenience and confidence with digital practice during COVID‐19 compared with before the pandemic. Respondents identified that specialist training (27%), funding for workplace devices (22%) and supportive leadership (19%) were most likely to facilitate sustained digital practice. Clinicians hoped for a blended approach going forward with technology enhancing existing best practice. Further prominent themes included digital accessibility for all and maintaining the increased opportunity for multidisciplinary working that videoconferencing has afforded. More service‐specific aspirations were bespoke technological solutions as well as parents/carers being able to engage remotely with school‐based provisions.

**Conclusions & Implications:**

During COVID‐19, paediatric SLTs’ recognition and acceptance of how technology can augment practice has accelerated, with particular value being placed on inclusivity, choice, training, resources, leadership and indication of effectiveness. These are important considerations to help guide the profession towards the long‐term digital enhancement of SLT services.

**WHAT THIS PAPER ADDS:**

**What is already known on the subject**

- The COVID‐19 pandemic led to an unprecedented interest in the use of technology across SLT practice. Studies are emerging nationally and internationally to identify the digital priorities of the SLT workforce, the areas of digital SLT provision that are thriving or lacking, and the evidence to guide clinical practice and service development.

**What this study adds to existing knowledge**

- This is the first UK‐wide COVID‐19 survey solely focusing on the digital practice of paediatric SLTs. The findings provide critical insights into SLT perception of how practice has been impacted in the early stages of the COVID‐19 pandemic, including digital trends that are service, condition and demographic specific. Tools and channels required to support sustained beneficial change are also discussed.

**What are the potential or actual clinical implications of this work?**

- This paper demonstrates the potential for digital solutions to enhance SLT practice, as long as implementation is guided by clinicians’ experiences and perspectives. The findings lay groundwork for service development work, such as the creation of training packages, updating of clinical guidelines and care pathways, and development of processes to ensure equitable allocation of evidence‐based resources.

## INTRODUCTION

### Context

On 30 January 2020, the World Health Organization's (WHO) Emergency Committee declared an international public health emergency following the outbreak of a novel coronavirus disease subsequently labelled COVID‐19 (WHO, 2020a, 2020b).

What followed was an urgency to reduce transmission rates through globally adopted measures that decreased individual mobility and increased social distancing (WHO, 2020c). In March 2020, the UK followed many countries in moving to a principle of ‘digital first’ (Parliament, House of Commons Chamber, 2020). This brought the use of technology into focus as a means to enable those in health, education and social care to continue offering their services. UK paediatric speech and language therapy (SLT) provision, which can straddle all three services, became part of this rapid shift towards greater digital practice.

Organization case studies show that introducing effective sustained digital care is a complex change extending far beyond adopting new technology (Greenhalgh et al., 2020). Multiple intertwined systems and routines are affected including clinical, privacy, security, accessibility and accountability. However, the rate of spread of COVID‐19 and the resulting urgency in introducing remote working measures has meant that the usual process of recording, monitoring and analysing that guides a major service change has so far been limited for paediatric SLT.

### Exploring the terminology

As the body responsible for international public health, WHO adopts the term ‘digital health’ to encompass electronic health, mobile health, telehealth and health data, among others. Sitting within the umbrella of digital health is a wide range of digital services and solutions, including videoconferencing, mobile health apps, integrated care delivery, clinical decision‐making support tools, nanotechnologies and artificial intelligence (WHO, 2020d).

In the UK, the Royal College of Speech and Language Therapy (RCSLT), which is the national professional body for SLTs, uses the term ‘telehealth’ to refer to what it describes as remote healthcare services. The RCSLT has adopted the WHO definition of telehealth, that is, the ‘delivery of health care services, where patients and providers are separated by distance’ (RCSLT, 2020a; WHO, 2016). A survey by the RCSLT identified that two‐thirds of SLTs prefer this term (RCSLT, 2020b), though the RCSLT acknowledged that telehealth may be used interchangeably with a range of terms including ‘telemedicine’, ‘telepractice’ and ‘teletherapy’ (RCSLT, 2020a). The American Speech–Language–Hearing Association (ASHA et al., 2021) has adopted telepractice with the rationale that this avoids the misperception that these services only apply to healthcare settings (ASHA, n.d.).

This paper will take a broad approach and explore all elements of digital transformation in UK paediatric SLT that have occurred as a result of the COVID‐19 pandemic.

### Current evidence base

In 2021, as a response to the widescale adoption of SLT remote working following the onset of COVID‐19, Law et al. (2021) published a rapid review of the existing reviews of digital practice for children with communication difficulties. This summative review adopted the Capability, Opportunity, Motivation and Behaviour (COM‐B) model of behaviour change (Michie et al., 2011) to critically analyse key elements of digital practice that have been reviewed empirically and generate broad recommendations for clinical implementation. This was done with the caveat that the evidence base is limited in scale and quality and generalization is influenced by a multitude of factors such as population, location, frequency and intervention provider.

The COM‐B framework is designed to map behaviour change in relation to three key drivers. First, capability, which is as an individual's physical and psychological capacity to engage in an activity. According to Law et al. (2021), there is little evidence in the literature of the SLT competencies required for effective digital practice. COM‐B identifies the second behaviour change driver as opportunity, breaking it down further into physical and social. Law et al. report the empirical benefits of physically adapting therapy materials for digital practice as well as improved outcomes when the quality of technical equipment is considered. With regards to social opportunity, increased involvement of parents, teachers and other relevant professionals offered by remote SLT sessions was found to impact positively on the child's communication outcomes, as was the opportunity for SLTs to view the child in more than one environment. The final behaviour change driver identified by the COM‐B model is motivation, which can be reflective in terms of an individual's evaluation of benefits and automatic, that is, the extent to which a behaviour becomes habitual. Whilst the literature indicates overall SLT motivation through acceptance of the benefits of digital practice, Law et al. acknowledge that clinicians in the studies reviewed may not be representative of the wider population, for example, in terms of access to technology. This has implications for the level of automatic motivation, given that the majority of research has focused on the introduction of technology as a novel SLT solution for a defined period of time whereas few studies have investigated the longitudinal impact of digital practice being embedded in routine practice.

With regards to existing studies of COVID‐19's impact on SLT services, the RCSLT has conducted a number of all‐member surveys (Chadd et al., 2021; RCSLT, 2020b, 2020c). Whilst response rates varied substantially between surveys, the findings overall indicate a significant increase in remote working measures following the onset of COVID‐19. The first COVID‐19 impact survey held by the RCSLT in April 2020 (RCSLT, 2020b) found that 60.7% (*n* = 330) of 544 respondents had moved to telephone consultations and 43.6% (*n* = 237) to video consultations. By November 2020, out of an admittedly far smaller sample of 147 respondents, 79.6% (*n* = 117) were using video consultations to some extent (RCSLT, 2020c).

The survey by the RCSLT in April 2020 also explored COVID‐19‐related service changes that respondents would like to see continue. Open‐text responses were grouped into themes that included ongoing online access to specialist training and peer support reinforced by the development of relevant clinical guidelines. SLTs also wished for continued flexibility in frequency and format of service delivery, particularly where it allowed increased family/parental involvement and multi‐professional collaboration. Areas of concerns resulting from the shift to remote working, which SLTs felt needed addressing, included unequitable service‐user access to technology and increased clinician anxiety resulting from a change in confidence of their role.

Outside the UK, international surveys are emerging that investigate digital practice during COVID‐19 across the SLT profession (Austria, Rettinger et al., 2021; Italy, Cacciante et al., 2021; Hong Kong, Fong et al., 2021; India, Aggarwal et al., 2020; and Canada, Macoir et al., 2021). Their overall scope complements the UK RCSLT surveys; however, in relation to the focus of this paper, only Fong et al. (2021) concentrate on paediatric clinicians who were surveyed in Hong Kong between February and March 2020. At the time, COVID‐19 had already resulted in remote working provision across Hong Kong given its proximity to the site of the pandemic onset. Of 135 respondents, 35.0% (*n* = 47) were providing clinical services remotely, of which 70.2% (*n* = 33) used videoconferencing and 51.1% (*n* = 24) used telephone. The majority, 72.3% (*n* = 34), reported adopting remote working for the first time following the onset of the pandemic.

Fong et al. (2021) used an open‐ended question format to ask participants what additional support was required for effective digital practice. Respondents’ replies were listed into overarching categories: this included the need for hardware such as tablets and smartphones as well as tools tailored to manage particular clinical needs, for example, dysphagia; another category was software that grouped videoconferencing platforms, parent education manuals, interactive apps/games and online database of resources; training was also identified as a distinct category of need for supporting the ongoing adoption of technology in practice. Training was explored further, and the survey reported that 60.0% (*n* = 81) of respondents had never had any digital practice training and 89.6% (*n* = 121) desired training.

By focusing on paediatric SLTs based in Hong Kong, Fong et al. (2021) explored factors relating to the use of technology in supporting children specifically, thereby making it a relevant source of comparison for a survey of paediatric SLT digital practice in the UK.

### The study's aims

This paper captures and reflects on the perception of UK paediatric SLTs in relation to their digital practice from pre‐ to during COVID‐19 and the implications moving forward.

## METHOD

See Appendix A in the additional supporting information for the full survey.

### Survey content

In the first section of the survey, paediatric SLTs were presented with background information and instructions. In the second section, they were asked to provide demographic information, including the geographical location of clinical practice, caseload composition, type of work setting and source of funding for the SLT service. The third section used closed questions to explore respondents’ perceived change in their digital practice pre‐ and during COVID‐19 and the factors facilitating their digital uptake. The final section gathered open‐text aspirations for the future role of technology in paediatric SLT.

### Survey development: Sections 3 and 4

Items for section 3 of the survey were informed by an initial audio‐recorded focus group discussion around digital practice with eight paediatric SLTs based at St George's University Hospitals NHS Foundation, London, who also piloted the survey. This method to guide survey content is in keeping with recommended practice for surveys when exploring areas with limited existing research (Nassar‐McMillan et al., 2010; O'Brien, 1993). Table 1 summarizes key participant characteristics in terms of caseload and pre‐COVID‐19 work setting.

**TABLE 1:** Focus group participant characteristics

**Caseload (*n*** **^a^**)		**Work setting pre‐COVID‐19 (*n*** **^a^**)	
Autism spectrum disorder	7	Mainstream primary schools	7
Developmental language disorder	7	Clinics	6
Language delay	6	Early years setting	6
Speech sound disorders	6	Special school/unit/resource base	3
Complex needs	3	Mainstream secondary schools	2
Learning disability	3		
Augmentative and Alternative Communication (AAC)	2		
Downs syndrome	2		
Hearing impairment	2		

*Note*: ^a^Out of eight SLT participants, some supported children across categories.

Inductive thematic analysis based on published guidance by Braun and Clarke (2006) was undertaken by the primary author to generate survey questions from a verbatim transcription of the focus group. The process involved reading the entire transcript to enable familiarization, then re‐reading and systematically coding areas of importance as they emerged from the data, that is, areas influencing clinicians’ digital views and experiences. These codes were clustered into themes and subthemes, which were validated through agreement with an experienced researcher and a focus‐group participant.

Two prominent themes generated from the focus group were that convenience and confidence with technology were required for digital uptake. These were divided into subthemes to provide a more defined list of facilitative factors.

The subthemes included the importance of supportive leadership:

With our manager we looked and researched together.

In addition, SLTs discussed the value of adopting a consistent model across a team:

We had tablets … for all community people we all had them and that was part of the mobile team working from top down to bottom up.

The value of specialist training was also highlighted:

There needs to be adequate training rather than just being expected to know.

As well as protected time to learn to deliver sessions:

If I had enough time to learn how to use in therapy myself then I could train people properly.

Funding for devices was also identified as a facilitator for digital uptake:

I was part of a funded project … every single therapist got their own iPad; you could download everything … like all the apps that you wanted … so, if it was available, we'd use it.

The final subtheme referred to the availability of service‐level guidance, policies and procedures:

We need clear guidance so there is consistency between clinicians.

These themes and subtheme guided section 3 of the survey, which began by asking clinicians to rate themselves pre‐ and during COVID‐19 using a five‐point Likert scale on their frequency, convenience and confidence of technology use. For frequency of technology use, respondents were also asked to identify through free‐text responses the type of technology used both pre‐ and during COVID‐19. Respondents were then presented with a list of factors facilitating the use of technology derived from the subthemes generated from the focus group interview and were asked to rate as most to least useful.

Section 4 marked the final phase of the survey and captured SLTs’ hopes for the future of digital practice. In keeping with comparative surveys of COVID‐19 SLT digital practice, this section was kept open ended to allow free‐text responses (Cacciante et al., 2021; Fong et al., 2021; Macoir et al., 2021; RCSLT, 2020b).

### Survey procedure and administration

The survey was designed to be completed online and was hosted on Qualtrics^®^ (2005), an online tool for generating, running and analysing surveys anonymously. The survey was advertised via the RCSLT's website, professional webinars, special interest groups, emails to service leads and social media (namely Twitter). Data were collected between 20 May and 30 October 2020.

### Survey data management

All survey data were stored and analysed anonymously using a restricted access portal. Demographic data were summarized as descriptive statistics. Quantitative data relating to digital practice and views were converted within the Qualtrics program into bar graphs.

### Survey analysis

Qualitative data consisting of clinicians’ open‐text responses on their hopes and aspirations for SLT digital practice underwent deductive framework analysis (Gale et al., 2013), with themes aligned to the typology of the COM‐B behaviour change model. As with inductive analysis, this involved familiarization with the dataset followed by systematic coding of areas of importance in the data. However, with a deductive framework approach, the codes are influenced by theory‐driven constructs, in this case the behaviour‐change components of the COM‐B model: capability, opportunity, motivation. A summary matrix created using Microsoft Excel clustered codes into themes and subthemes through iterative triangulation between the quotations, the emerging themes and the theoretical concepts represented within the COM‐B model. The second author led the qualitative analysis, which was then reviewed by the primary author; where agreement could not be reached consensus was sought from the third author. A thematic map was generated to visually represent the association between the themes, and this was complemented by a detailed overarching interpretation of the thematic patterns reinforced by direct quotations.

The COM‐B model was selected for the analytical framework because it provides a systematic method of considering paediatric SLTs’ views on digital practice according to areas that are important for effective behaviour change, that is, the sustained adoption of technology by clinicians, thus supporting the clinical translation of research (Michie et al., 2011). Furthermore, as the COM‐B model has been adopted by Law et al. (2021) to define behaviour‐change drivers in their summative review of digital intervention reviews for children with communication difficulties, it enables a comparison of the survey findings with the existing research corpus.

### Ethical considerations

Approval for this study was obtained from the St George's University Hospitals NHS Foundation Trust's Research Ethics Committee (SGREC17.0026).

## RESULTS

### Demographics

In total, 432 paediatric SLTs responded to the online survey; eight practiced internationally, leaving 424 responses from UK‐based clinicians, which were analysed for this paper. Table 2 provides a summary of respondent characteristics. SLTs were asked to select their demographic characteristics from a list of options, including an ‘other—please specify’ section, and could select all that applied to them.

### SLTs’ perception of their digital practice before and during the COVID‐19 pandemic

**FIGURE 1:**
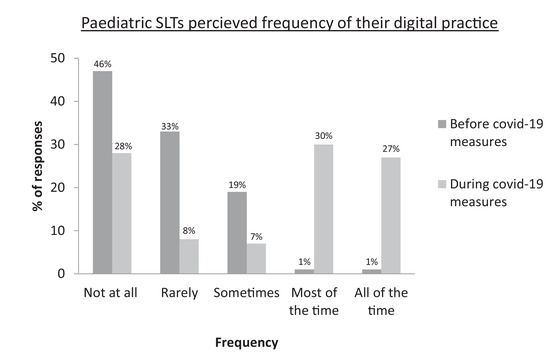
Paediatric SLTS’ perceived frequency of using technology in practice before and during COVID‐19

**FIGURE 2:**
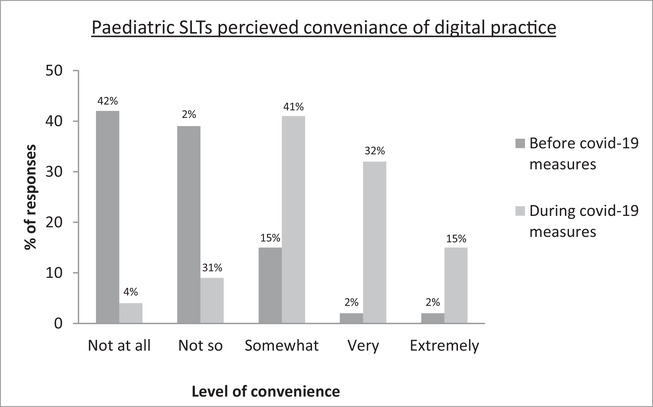
Paediatric SLTS’ perceived convenience of using technology in practice before and during COVID‐19

**FIGURE 3:**
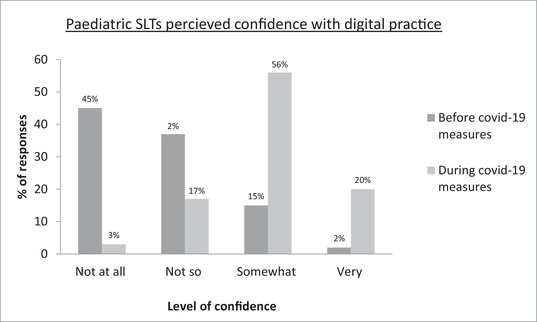
Paediatric SLTS’ perceived confidence with using technology in practice before and during COVID‐19

Respondents were asked to rate their frequency (Figure 1), convenience (Figure 2) and confidence (Figure 3) in relation to their use of technology in SLT before and during COVID‐19 measures; findings suggest a positive trend in all three areas.

For the question of digital frequency, SLTs were asked to identify, through free‐text response, which technology they used before and during COVID‐19 measures, with the option to submit multiple responses (Table 3). Most noticeable is the reported increase in use of video calls during COVID‐19, with a greater reference to types of videoconferencing technology when compared with the time pre‐pandemic. The difference appears to reflect the increase in uptake and range of remote‐working practice in response to COVID‐19 measures. Respondents also indicated a decrease in the use of iPads and a comparable increase in laptop use. This could demonstrate the preferred hardware for remote working, though more information would need to be gathered.

**TABLE 2:** Survey respondent characteristics

**Demographic measure**		**Percentage of respondents from a total of 424 (*n*). Multiple responses could be selected for each demographic measure**	
		**Response rate**	
*Country of practice*		**Estimated UK paediatric SLTs** **^a^**	**Rate**
England	81.4% (345)	9 199	3.8%
Scotland	7.3% (31)	773	4.0%
Wales	6.8% (29)	411	7.1%
Northern Ireland	4.5% (19)	475	4.0%
*Caseload*			
Autism spectrum disorder	67.5% (286)		
Developmental language disorder	60.4% (256)		
Speech sound disorders	56.6% (240)		
Language delay	49.5% (210)		
Complex needs	38.0% (161)		
Learning disability	36.8% (156)		
Dysphagia	24.8% (105)		
Downs syndrome	22.9% (97)		
AAC	23.3% (99)		
Hearing impairment	12.7% (54)		
Dysfluency	7.1% (30)		
Cleft palate	6.8% (29)		
Visual impairment	5.7% (24)		
Other (please specify)	1.7% (7) = 1.2% (5) selective mutism and 0.5% (2) cerebral palsy		
*Work setting (pre‐COVID‐19)*			
Mainstream primary schools	64.9% (275)		
Clinics	49.1% (208)		
Special school/unit/resource base	33.3% (141)		
Early years setting	33.0% (140)		
Mainstream secondary schools	30.1% (130)		
Client's home	27.1% (116)		
Your home/office	12.0% (51)		
Other (please specify)	7.0% (30) = 6.1% (26) hospital, 0.7% (3) college, 0.2% (1) Child and Adolescent Mental Health Services (CAMHS)		
Residential setting	1.7% (7)		
*Source of funding*			
National Health Service (NHS)	83.7% (355)		
Local authority	22.4% (95)		
Private/independent	10.4% (44)		
Other (please specify)	4.2% (18) = 3.5% (15) schools, 0.7% (3) public healthcare Ireland		
Charity	2.6% (11)		

*Note*: ^a^Extrapolated from data obtained from RCSLT (2021a).

**TABLE 3:** Types of technology reported to be used by paediatric SLTs before and during COVID‐19

	**Percentage of respondents (*n*)**	
**Type of response: Multiple free‐text**	**Before COVID‐19**	**During COVID‐19**
**responses were allowed**		
*Hardware*		
iPad	27.1% (115)	5.2% (22)
Laptop	3.5% (15)	21.9% (93)
Telephone	2.4% (10)	11.8% (50)
*Software*		
Video clips	25.9% (110)	
Apps	20.5% (87)	28.1% (119)
Video call	2.8% (12)	51.2% (217)
MS Teams	0.9% (4)	2.8% (12)
WhatsApp	0.5% (2)	
Bluejeans	0.2% (1)	1.9% (8)
Website		28.3% (120)
Attend anywhere		20.8% (88)
Zoom		16.0% (68)
Skype		4.2% (18)
Teletherapy		3.3% (14)
Facetime		2,8% (12)
NHS near me		1.2% (5)
Webex		1.2% (5)

### Factors facilitating the uptake of technology

Respondents were then presented with a list of activities that support digital practice which they were asked to rank in order of benefit (Table 4).

**TABLE 4:** Activities identified by paediatric SLTs as facilitating the uptake and use of digital practice

**Factors facilitating digital practice in paediatric SLT**	**% (n)**
Specialist training, e.g., webinars	27.4% (116)
Funding for workspace devices (laptops, smartphones, etc.)	21.9% (93)
Supportive leadership	19.1% (81)
Protected time to work out how to deliver sessions	13.9% (59)
Service level guidance, policies and protocols	8.7% (37)
Whole‐team approach	8.7% (37)

### Hopes and aspirations for the use of technology in SLT

**FIGURE 4:**
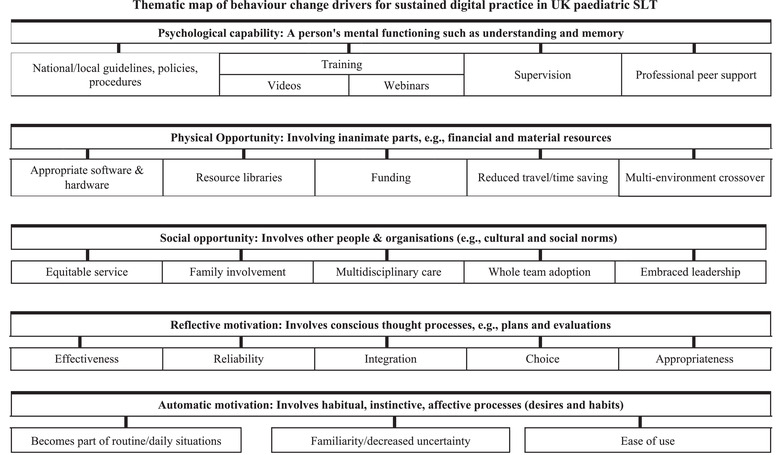
Thematic map of behaviour change drivers for sustained digital practice in UK Paediatric Speech and Language Therapy. [Correction added on 14 September after online publication: The title of Figure 4 is updated in this new version]

This section generated 410 open‐text responses which were analysed using the COM‐B model to identify behaviour change drivers as summarized below. In addition, a thematic map has been generated to visually summarize the findings (Figure 4).

### Capability

This survey suggests that increased exposure to technology following COVID‐19 onset has led to paediatric SLTs desiring improved digital knowledge and expertise. Preferences for support included formal measures such as policies, procedures and training materials with flexible access, for example, ‘how‐to videos’ and webinars as well as more interactive ongoing supervision and peer‐support routes. It was proposed that this would lead to a more informed and aligned workforce:

“I would like clearer guidance on how to use teletherapy effectively … there feels like a lack of consistency between clinicians and guidance on how to carry out these sessions”.

### Opportunity: Physical

Respondents described how the success of digital practice was dependent on the extent to which the technology met their needs and those of the client group.

It was expressed that as SLT input requires specialized judgement of communication and/or feeding skills, the quality of the technology could highly influence a session. Furthermore, the age group being supported meant engaging tools were needed that allowed clinicians to model activities that the child and carer or educator could replicate:

“To be granted platforms with improved functionality, i.e., being able to see the child clearly whilst sharing a slideshow containing a game”.

The high‐level technological requirements for digital practice led to many respondents discussing the need for proper investment in resources:

“Teletherapy sessions require a lot of preparation. I hope that an ‘online teletherapy resource database’ can be created of activities/assessments that SLTs can instantly download to use with clients (e.g., for different clinical areas)”.

“That appropriate funding is allocated to purchase suitable equipment to continue the work started”.

The opportunity for digital tools to reduce some of the logistical issue with accessing face‐to‐face SLT sessions, was explored:

“To continue reducing barriers for clients e.g., travel to and from clinic, and therapists' travel time between settings”.

Finally, multi‐environmental crossover was noted as a strength of remote practice that respondents valued:

“Normally the majority of our input is delivered within the child's education setting … it would be beneficial to also see the child in their home environment … some will have different strengths and needs in different settings”.

### Opportunity: Social

The role of technology with regards to family involvement was considered. There was reservation that clinical interaction would not be as effective if only delivered remotely:

“It can be difficult to build that initial rapport with the child/family purely over telehealth … I would prefer to see them first face to face, then to move to telehealth”.

However, the opportunity for digital practice to provide the flexibility to offer services that suit family preferences including through multidisciplinary working was also expressed:

“Attendance at [school] coffee mornings, parent's evening etc. is variable and can be poor. Telehealth has enabled better links between professional colleagues, therapists and parents than I have ever known (have worked in special schools since 2007) which I hope will continue”.

Respondents felt that services needed to address digital exclusion better, whether caused by socio‐economic status, clinical need or connectivity issues such as in rural areas:

“Currently some families don't have access to technology, or English Additional Language/British Sign Language interpreters; creating a divide between those that have the technology and those that don't”.

The complexity of supporting vulnerable families was also covered both in terms of potential disengagement and also a difficulty in identifying nuances of behaviour and interaction:

“Several families who may have safeguarding concerns do not engage in video consultations/ phone calls as much, I hope that this area is prioritised”.

“You are one step removed from the situation. You have to be more intentionally probing with questions as it is more difficult to pick up cues that might alert you to an issue”.

Finally, respondents identified the need for clear leadership and team‐wide approaches for collaborative exploration of problems and solutions:

“I hope SLT management are more proactive in providing opportunities for colleagues to share what they are doing/what works/doesn't work—at present our Trust has not been good at doing this, so all colleagues are busy reinventing their own wheels”.

### Motivation: Reflective

Respondents wanted to feel sufficiently informed so that they could make reasoned judgements about digital practice. Factors that influenced decision‐making included the security of the digital solution, comparability to face‐to‐face methods and empirical indications of effectiveness:

“That information surrounding telehealth remains balanced and isn't driven by people sharing just what has worked, but what hasn't worked as well”.

The increased choice in service‐delivery methods that technology offered was consistently of importance to clinicians:

“That we can use a blended approach within our service to get the best of both worlds”.

“That children who benefit from telehealth more than face‐to‐face will have ongoing access to this beyond this pandemic (e.g., where, for example, two shorter sessions a week are better than one longer one and this is now feasible with telecare)”.

The perceived appropriateness of digital practice was varied. Some felt that the use of remote technology contradicted the measures required to meet the communication and feeding needs of the populations supported by paediatric SLTs:

“That it is not used. To not be in the same room as the patient and attempt to bring about outcomes through use of yet more screen time, when this is the very thing that we advise to reduce in order to enhance communication, it is … clinically inappropriate”.

“I work solely in dysphagia, I am extremely concerned about the safety of working via telehealth & don't feel it is at all appropriate for working with aspiration risk unless the child & carer is very well known & a low risk”.

Whilst others considered technology as serving as a facilitator to supporting the same clinical populations:

“That therapists will start to recognise the benefits of digital service delivery and more importantly the possibilities and opportunities working with technology can offer our clients”.

“Almost all of my telehealth is in dysphagia. I hope that it continues to be a very useful tool in reviewing and monitoring changes and progress and this it will reduce non‐attendance and to a certain extent non‐compliance”.

The range of experiences and opinions in relation to digital practice indicates the need to consider multiple viewpoints in order to gain a reflective picture and reduce the risk of bias.

### Motivation: Automatic

The SLTs surveyed desired technology that was convenient and accessible in order for digital tools to become embedded and automatic in clinical practice. Respondents were motivated by the potential for technology to enable children alongside their carers and partner professionals to embed communication and feeding support habitually as and when required:

“That it will further facilitate carryover of strategies into everyday routines and activities for example using items available to families at home”.

The level of acceptance of digital practice appeared closely linked to opportunities to use and familiarize with the medium:

“Not sure—still haven't been able to use it due to NHS redeployment—but the idea of it scares me quite a bit—really not sure how I will be able to use it for assessment in many different cases”.

## DISCUSSION

This study gives an insight into the influence of the COVID‐19 pandemic on the digital perception of a sizable sample of UK paediatric SLTs in the months following the introduction of remote working measures. With a participation rate reaching 3–7% of the Paediatric SLT workforce, this survey has the highest response rate of UK and international surveys of the impact of COVID‐19 to date.

The results indicate that clinicians not only report a marked shift in their digital practice over a short period of time, but there are indications that the learning could lead to fundamental changes in the role of technology within SLT services.

### Nature of digital transformation

This paper complements existing national and international COVID‐19 SLT surveys (Aggarwal et al., 2020; Fong et al., 2021; Macoir et al., 2021; RCSLT, 2020b, 2020c) by identifying a perceived increase in the frequency of digital practice and additionally identifies a corresponding acceleration in perceived digital convenience and confidence amongst paediatric SLTs.

As pandemic measures are lifted, there appears real potential to combine the therapeutic use of technology that appeared common pre‐pandemic with the focus on remote‐access tools during the pandemic in order to provide a more blended approach.

### Facilitating digital practice

Respondents considered specialist training as most helpful in supporting digital practice, strongly followed by access to devices then supportive leadership. The importance of training aligns with the results from the RCSLT member surveys (RCSLT, 2020b, 2020c) and as the leading national body representing SLTs, this has influenced RCSLT guidance and support. They have identified that the widescale adoption of technology in practice requires informed clinicians who are working to provide the necessary training to upskill the workforce (RCSLT, 2020c, 2021c). There is less direction in terms of funding to support digital practice and this could lead to variation in how and what technology is commissioned, particularly given the plethora of funding sources for SLT provision such as health services, local government, schools, charities and the private sector (Table 1) (see also RCSLT, 2020b).

As evidenced by the spread of responses, a focus on training, technology and leadership is not sufficient to embed SLT digital practice. Respondents also selected protected time, localized guidance and peer support as facilitating factors. This indicates that the service changes required for digital transformation are multifaceted and require combined consideration for clarity and cohesion, as is advocated by national and international digital practice guidance that have emerged following COVID‐19 onset (RCSLT, 2020a; WHO, 2020e).

### The future of digital in paediatric SLT

By capturing the technological hopes and aspirations of practising UK paediatric SLTs, direct insight can be gained into what is and is not working at a clinical level. This helps identify the support clinicians require to provide the best possible care under the constraints of COVID‐19 remote working as well as steps to ensure that benefits of technology are sustained beyond the pandemic.

The consistency with which digital proficiency was desired by SLTs in this survey and the lack in the current evidence base of the skills required for effective practice (Law et al., 2021) highlights not only a research need but also the importance of guidance from other sources such as the UK Allied Health Professionals Digital Competency Framework (NHS Health Education England, 2020).

Survey respondents valuing access to appropriate digital tools aligns with empirical findings. This includes a systematic review of SLT digital interventions that found 75.0% of 103 selected studies reported technological limitations as impacting effectiveness and recommended user data to improve software (Molini‐Avejonas et al., 2015). The implications are that for digital SLT solutions to be fit for purpose they need to be designed and developed with the involvement of clinicians as well as the children and carers that the clinician's serve, the latter being a voice which is increasingly emerging in COVID‐19 research (Lam et al., 2021; RCSLT, 2021b). This collaborative stakeholder approach is strongly advocated by the RCSLT which has partnered with the National Institute of Health Research's Centre for Engagement and Dissemination to learn from SLT service users and deliverers about their digital priorities (Chadd et al., 2020).

Increased collaborative opportunities during remote working whilst reported in this survey are not replicated in the Hong Kong digital practice survey, which is the only other COVID‐19 study focusing solely on paediatric SLT (Fong et al., 2021). Existing research has demonstrated that the impact of a child's communication and/or feeding needs and the subsequent approach to SLT management can be influenced by a range of social, cultural, political and disability‐related factors (Babatsouli, 2021). Whilst beyond the scope of this paper, it would be useful for future research to consider whether there are implications in terms of digital provisions.

Another explanation for this difference in survey findings is timing. The Hong Kong survey was conducted early in the pandemic whilst the country was still in lockdown. In contrast, this UK survey spanned both the first lockdown when schools closed and remote home‐based therapy was introduced through to subsequent easing when SLT provision generally returned to being school based, thus providing first‐hand experience of the potential for technology to bridge the home–school divide. This feeds into a wider point that the pace of COVID‐19 digital transformation makes the applicability of the survey findings dependent on the specific public health measures being implemented at the time of data collection. Though, as the focus of this paper is to explore the underlying behaviour change elements of digital transformation, its relevancy does have longevity beyond the immediate pandemic situation.

Restricted opportunities for digital access due to lack of remote interpreting provisions is mentioned in this paper but not in other surveys of COVID‐19 SLT. This is concerning given the research demonstrating improved communication outcomes when the child's home language is considered during digital practice (Mashima & Doarn, 2008). The issue corresponds with wider digital exclusion and disengagement concerns, and whilst these have been raised in both this and other surveys of COVID‐19 SLT practice (Cacciante et al., 2021; Fong et al., 2021; RCSLT, 2020b; Rettinger et al., 2021), there is a lack of SLT research emerging from COVID‐19 that focuses on how the inequalities can be addressed.

In this survey, SLT expressed reservations about the comparability of digital practice to face‐to‐face provision, a theme replicated in most COVID‐19 SLT surveys (Aggarwal et al., 2020; Fong et al., 2021; Macoir et al., 2021; Rettinger et al., 2021; RCSLT, 2020b, 2020c). Interestingly, the research has demonstrated fidelity, validity and reliability of certain remote SLT management activities compared with in‐person practice, including some non‐standardized and standardized assessments and targeted interventions for speech, language and social skills (Grogan‐Johnson et al., 2010; Shire et al., 2020; Stavropoulos et al., 2022; Taylor et al., 2014). This perhaps indicates a clinical disconnect with the empirical data and highlights the need for clinical–academic collaboration across all stages of research including design, implementation, analysis and dissemination.

## LIMITATIONS

As this survey was in response to the rapid progression of COVID‐19 and subsequent changes to paediatric SLT digital practice, respondent's digital views and experiences before the pandemic were captured retrospectively and compared with their current ratings. This method has the risk of recall bias and results should therefore be interpreted with caution.

Another limitation is that both recruitment and survey completion occurred digitally, potentially attracting a greater proportion of respondents with a positive attitude towards technology. In addition, although this survey had a high response rate relative to comparable COVID‐19 digital SLT practice surveys, the findings still only represent the responses at one point in time of a subsection of all UK paediatric SLTs, and even within this sample a broad range of views were expressed. As a result, findings should not be considered a stable consensus of UK paediatric SLTs’ perception of digital practice. Rather, the views of a proportion of the population during a period of swift change need to be considered alongside the broader empirical landscape to allow a robust interpretation.

With regards to the use of focus group findings to guide survey instrument development, whilst this provides a replicable and empirically reasoned methodology, a limitation is the potential influence of the researcher on the interpretation of the data and the survey questions generated, particularly given that the majority of questions were closed‐ended (Saris & Gallhofer, 2014). In line with recommended practice (Morse, 2015; Shenton, 2004), mitigations included presenting data extracts to demonstrate consistency in data analysis and interpretation; validation through consensus and assessing credibility by comparing it with survey instruments in comparable studies (e.g., Fong et al., 2021; RCSLT, 2020b, 2020c).

## CONCLUSIONS

This paper provides a snapshot of both the rapid pace and extensive scale of digital transformation arising during the first year of the COVID‐19 pandemic in the context of UK paediatric SLT practice. This has led to increased awareness amongst clinicians of the challenges and opportunities afforded by technology and subsequent recognition of SLT‐specific measures required for sustainable and responsive digital support systems.

## CONFLICT OF INTEREST

The authors report no conflicts of interest. The authors alone are responsible for the content and writing of the paper.

## Data Availability

The data that support the findings of this study are available from the corresponding author upon reasonable request.

## APPENDIX A SURVEY OF THE IMPACT OF COVID‐19 ON DIGITAL PRACTICE IN UK PAEDIATRIC SPEECH AND LANGUAGE THERAPY

### Background information

The current context of COVID‐19 social‐distancing has provided an **unprecedented** opportunity for paediatric Speech and Language Therapists (SLT's) to utilise technology to support children and families on their caseloads. **We would love to gather your views to gain an understanding of SLT opinion and practice** in relation to the use of technology when providing care.

**All responses will remain anonymous and confidential**. Please answer all questions and write comments/ suggestions in the spaces provided.

Thank you for your help!

Approval has been obtained from St George's University Hospitals Foundation Trust's Research Ethics Committee (SGREC17.0026) and the Health Research Authority.

Q1 Where are you located when practising as an SLT? (Tick which apply to you)

○ England
○ Northern Ireland
○ Scotland
○ Wales
○ International (Please state the country in the box below)

______________________________________

Q2 What is your caseload made up of? (Tick which apply to you)

○ Autism Spectrum Disorder
○ AAC
○ Cleft Palate
○ Complex Needs
○ Developmental Language Disorder
○ Dysfluency
○ Downs Syndrome
○ Learning Disability
○ Language Delay
○ Hearing Impairment
○ Speech Sound Disorders
○ Visual Impairment
○ Other (please specify)

______________________________________

Q3 Which setting do you usually work in, that is, pre‐COVID‐19? (Tick which apply to you)

○ Client's Home
○ Clinics
○ Early Years Setting (e.g., nursery/children's' centre)
○ Mainstream Primary School
○ Mainstream Secondary School
○ Residential Setting
○ Special School/Unit/Resource Base
○ Your Home/Office

o Other (please specify)
__________________________________

Q4 How is your SLT work funded? (Tick which apply to you):

○ Charity
○ NHS
○ Local Authority
○ Private/Independent
○ Other (please specify)

______________________________________

Q5 **Before** COVID ‐19 measures, how often did you use technology to deliver SLT?

○ Not at all
○ Rarely
○ Sometimes
○ Most of the time
○ All of the time

Q6 Please identify which type/s of technology in the comments box below:

_____________________________________________

Q7 **During** COVID ‐19 measures, how often do you use technology to deliver SLT?

○ Not at all
○ Rarely
○ Sometimes
○ Most of the time
○ All of the time

Q8 Please identify which type/s of technology in the comments box below:

_____________________________________________

Q9 **Before** COVID ‐19 measures, how confident did you feel about using technology to deliver SLT?

○ Extremely confident
○ Very confident
○ Somewhat confident
○ Not so confident
○ Not at all confident

Q10 **During** COVID ‐19 measures, how confident do you feel about using technology to deliver SLT?

○ Extremely confident
○ Very confident
○ Somewhat confident
○ Not so confident
○ Not at all confident

Q11 **Before** COVID ‐19 measures, how convenient did you find the use of technology to deliver SLT?

○ Extremely convenient
○ Very convenient
○ Somewhat convenient
○ Not so convenient
○ Not at all convenient

Q12 **During** COVID ‐19 measures, how convenient do you find the use of technology to deliver SLT?

○ Extremely convenient
○ Very convenient
○ Somewhat convenient
○ Not so convenient
○ Not at all convenient

Q13 What do you think would **help you** to use technology in SLT?

Rate the following statements by dragging and dropping each statement (**1 = most useful, 6 = least useful**)

______ Funding for workplace devices
______ Protected time to work out how to deliver sessions
______ Service level guidance, policies, and protocols
______ Specialist training (e.g., webinars)
______ Supportive leadership
______ Whole team approach

Q20 What is your biggest hope or aspiration for the use of technology in paediatric SLT practice?

_____________________________________________

We thank you for your time spent taking this survey.

## References

[jlcd12750-bib-0001] Aggarwal, K. , Patel, R. & Ravi, R. (2020) Uptake of telepractice among speech–language therapists following COVID‐19 pandemic in India. Speech, Language and Hearing, pp. 1–7.

[jlcd12750-bib-0002] AMERICAN SPEECH HEARING ASSOCIATION (ASHA) . (n.d.) Professional issues: Telepractice. [online]. Available: www.asha.org/practice‐portal/professional‐issues/telepractice/ [Accessed 10 March 2021].

[jlcd12750-bib-0003] Babatsouli, E. (2021) Diversity Considerations in Speech and Language Disorders: A Focus on Training. In Damico, J. S. , Müller, N. and Ball, M. J. (Eds) The Handbook of Language and Speech Disorders, Second Edition. Wiley Online Library. pp 33–52. 10.1002/9781119606987.ch2

[jlcd12750-bib-0004a] Cacciante, L. , Cieślik, B. , Rutkowski, S. , Rutkowska, A. , Kacperak, K. , Kuligowski, T. & Kiper, P. (2021) Feasibility, acceptability and limitations of speech and language telerehabilitation during COVID‐19 lockdown: a qualitative research study on clinicians' perspectives. *Healthcare* 9(11), 1503. MDPI. 10.3390/healthcare9111503 PMC8618578 34828549

[jlcd12750-bib-0005] Chadd, K.E. , Kulkarni, A.A. & Longhurst, L.M. (2020) Involving individuals with developmental language disorder and their parents/carers in research priority setting. Journal of Visualized Experiments, (160), e61267. https://www.jove.com/t/61267/involving-individuals-with-developmental-language-disorder-their 10.3791/61267 32568234

[jlcd12750-bib-0006] Chadd, K. , Moyse, K. & Enderby, P. (2021) Impact of COVID‐19 on the speech and language therapy profession and their patients. Frontiers in Neurology, 12, 96. 10.3389/fneur.2021.629190 PMC7930219 33679590

[jlcd12750-bib-0007] Fairweather, G.C. , Lincoln, M.A. & Ramsden, R. (2016) Speech language pathology teletherapy in rural and remote educational settings: decreasing service inequities. International Journal of Speech–Language Pathology, 18, 592–602. 10.3109/17549507.2016.1143973 27063692

[jlcd12750-bib-0008] Fong, R. , Chun Fung, T. & Yan Yiu, O.I. (2021) The implementation of telepractice in speech language pathology in Hong Kong during the COVID‐19 pandemic. Telemedicine and e‐Health, 27(1), 30–38. 32667859 10.1089/tmj.2020.0223

[jlcd12750-bib-0009] Gale, N.K. , Heath, G. , Cameron, E. , Rashid, S. & Redwood, S. (2013) Using the framework method for the analysis of qualitative data in multi‐disciplinary health research. BMC Medical Research Methodology, 13(1), 1–8. 24047204 10.1186/1471-2288-13-117 PMC3848812

[jlcd12750-bib-0010] Greenhalgh, T. , Wherton, J. , Shaw, S. & Morrison, C. (2020) Video consultations for covid‐19. BMJ, 368, m998. 32165352 10.1136/bmj.m998

[jlcd12750-bib-0011] Grogan‐Johnson, S. , Alvares, R. , Rowan, L. & Creaghead, N. (2010) A pilot study comparing the effectiveness of speech language therapy provided by telemedicine with conventional on‐site therapy. Journal of Telemedicine and Telecare, 16(3), 134–139. 20197354 10.1258/jtt.2009.090608

[jlcd12750-bib-0012] Lam, J.H.Y. , Lee, S.M.K. & Tong, X. (2021) Parents’ and students’ perceptions of telepractice services for speech–language therapy during the COVID‐19 pandemic: survey Study. JMIR Pediatrics and Parenting, 4(1), e25675. 33449909 10.2196/25675 PMC7850632

[jlcd12750-bib-0013] Law, J. , Dornstauder, M. , Charlton, J. & Gréaux, M. (2021) Tele‐practice for children and young people with communication disabilities: employing the COM‐B model to review the intervention literature and inform guidance for practitioners. International Journal of Language & Communication Disorders, 56(2), 415–434. 33522068 10.1111/1460-6984.12592

[jlcd12750-bib-0013a] Macoir, J. , Desmarais, C. , Martel‐Sauvageau, V. & Monetta, L. (2021) Proactive changes in clinical practice as a result of the COVID‐19 pandemic: Survey on use of telepractice by Quebec speech‐language pathologists. International Journal of Language & Communication Disorders, 56(5), 1086–1096. 34455652 10.1111/1460-6984.12669 PMC8652496

[jlcd12750-bib-0014] Mashima, P.A . & Doarn, C.R. (2008) An overview of telehealth activities in speech–language pathology. Telemedicine and eHealth, 14, 1101–1117. 10.1089/tmj.2008.0080 19119834

[jlcd12750-bib-0015] Michie, S. , Van Stralen, M.M. & West, R. (2011) The behaviour change wheel: a new method for characterising and designing behaviour change interventions. Implementation Science, 6, 42 21513547 10.1186/1748-5908-6-42 PMC3096582

[jlcd12750-bib-0016] Molini‐Avejonas, D. , Rondon‐Melo, S. , Amato, C. , Amato, H. & Samelli, A. (2015) A systematic review of the use of telehealth in speech, language and hearing sciences. Journal of Telemedicine and Telecare, 21(7):367–376. 10.1177/1357633/15583215. 26026181

[jlcd12750-bib-0017] Nassar‐McMillan, S.C. , Wyer, M. , Oliver‐Hoyo, M. & Ryder‐Burge, A. (2010) Using focus groups in preliminary instrument development: expected and unexpected lessons learned. The Qualitative Report, 15(6), 1621.

[jlcd12750-bib-0018] NHS HEALTH EDUCATION ENGLAND . (2020) UK Allied Health Professionals Digital Competency Framework . https://www.hee.nhs.uk/sites/default/files/Development%20of%20a%20digital%20competency%20framework%20for%20UK%20AHPs.pdf

[jlcd12750-bib-0019] O'Brien, K. (1993) Improving survey questionnaires through focus groups. Successful Focus Groups: Advancing the State of the Art, 156, 105–117.

[jlcd12750-bib-0020] Parliament, House Of Commons Chamber . (2020) Coronavirus. London: The Stationery Office.

[jlcd12750-bib-0021] Qualtrics . (2005) Qualtrics, Provo Utah USA. Version used: December 2020–March 2021. [online]. Available at: https://www.qualtrics.com [Accessed 31 March 2021].

[jlcd12750-bib-0022] Rettinger, L. , Klupper, C. , Werner, F. & Putz, P. (2021) Changing attitudes towards teletherapy in Austrian therapists during the COVID‐19 pandemic. Journal of Telemedicine and Telecare. 10.1177/1357633X20986038 PMC10195684 33430678

[jlcd12750-bib-0023] ROYAL COLLEGE OF SPEECH AND LANGUAGE THERAPISTS . (2020a) Telehealth Guidance. [online] Available at: https://www.rcslt.org/members/delivering‐quality‐services/telehealth/telehealth‐guidance/ [Accessed 8 Jan 2021].

[jlcd12750-bib-0024] ROYAL COLLEGE OF SPEECH AND LANGUAGE THERAPISTS . (2020b) Impact of the COVID‐19 pandemic on the speech and language therapy profession (Initial survey: April 2020). Available at [Accessed 8 Jan 2021].

[jlcd12750-bib-0025] ROYAL COLLEGE OF SPEECH AND LANGUAGE THERAPISTS . (2020c) Technology and digital health survey findings: November 2020. Internal report. Unpublished.

[jlcd12750-bib-0026] ROYAL COLLEGE OF SPEECH AND LANGUAGE THERAPISTS . (2021a) Impact of the pandemic on SLT provision (Follow up survey: February 2021)—Respondent data. Survey available at https://www.rcslt.org/learning/covid‐19/rcslt‐survey‐impact‐of‐the‐pandemic‐on‐service‐provision/section‐3 Respondent data available by contacting info@rcslt.org

[jlcd12750-bib-0027] ROYAL COLLEGE OF SPEECH AND LANGUAGE THERAPISTS . (2021b) Building back better: Speech and language therapy services after COVID‐19 [online] Available at: https://www.rcslt.org/get‐involved/building‐back‐better‐speech‐and‐language‐therapy‐services‐after‐COVID‐19/ [Accessed on 12 March 2021].

[jlcd12750-bib-0028] ROYAL COLLEGE OF SPEECH AND LANGUAGE THERAPISTS . (2021c) Technology Guidance [online] Available at: https://www.rcslt.org/members/delivering‐quality‐services/technology/technology‐guidance/ [Accessed on 14 November 2021].

[jlcd12750-bib-0029] Saris, W.E. & Gallhofer, I.N. (2014) Design, evaluation, and analysis of questionnaires for survey research. John Wiley & Sons.

[jlcd12750-bib-0030] Shire, S.Y. , Baker Worthman, L. , Shih, W. & Kasari, C. (2020) Comparison of face‐to‐face and remote support for interventionists learning to deliver JASPER intervention with children who have autism. Journal of Behavioral Education, 29, 317–338. 10.1007/s10864-020-09376-4

[jlcd12750-bib-0031] Stavropoulos, K.K.‐M. , Bolourian, Y. & Blacher, J. (2022) A scoping review of telehealth diagnosis of autism spectrum disorder. PLoS One, 17(2), e0263062. 10.1371/journal.pone.0263062 35143494 PMC8830614

[jlcd12750-bib-0032] Taylor, O.D. , Armfield, N.R. , Dodrill, P. & Smith, A.C. (2014) A review of the efficacy and effectiveness of using telehealth for paediatric speech and language assessment. Journal of Telemedicine and Telecare, 20(7), 405–412. 25400002 10.1177/1357633X14552388

[jlcd12750-bib-0033] West, R. & Michie, S. , (2020) A brief introduction to the COM‐B Model of behaviour and the PRIME Theory of motivation [v1]. Qeios, 10.32388/WW04E6

[jlcd12750-bib-0034] WORLD HEALTH ORGANISATION . (2016) Global Health Observatory (GHO) data: Telehealth [online]. Available at: https://www.who.int/gho/goe/telehealth/en/ [Accessed on 2 March 2021].

[jlcd12750-bib-0035] WORLD HEALTH ORGANISATION . (2020a) Transcript: WHO Emergencies Coronavirus Emergency Committee Second Meeting—30 January 2020 [online] Available at: https://www.who.int/docs/default‐source/coronaviruse/transcripts/ihr‐emergency‐committee‐for‐pneumonia‐due‐to‐the‐novel‐coronavirus‐2019‐ncov‐press‐briefing‐transcript‐30012020.pdf?sfvrsn=c9463ac1_2 [Accessed 12 June 2020].

[jlcd12750-bib-0036] WORLD HEALTH ORGANISATION . (2020b) WHO Situation Report: 11 February 2020 [online]. Available at: https://www.who.int/docs/default‐source/coronaviruse/situation‐reports/20200211‐sitrep‐22‐ncov.pdf?sfvrsn=fb6d49b1_2 [Accessed 12 June 2020].

[jlcd12750-bib-0037] WORLD HEALTH ORGANISATION . (2020c) Coronavirus disease (COVID‐19) advice for the public, 2020 [online]. Available: https://www.who.int/emergencies/diseases/novel‐coronavirus‐2019/advice‐for‐public [Accessed 12 June 2020].

[jlcd12750-bib-0038] WORLD HEALTH ORGANISATION . (2020d) Digital health: transforming and extending the delivery of health services. [online] Available: https://www.who.int/emergencies/diseases/novel‐coronavirus‐2019/advice‐for‐public [Accessed 28 March 2020].

[jlcd12750-bib-0039] WORLD HEALTH ORGANIZATION . (2020e) Implementing telemedicine services during COVID‐19: guiding principles and considerations for a stepwise approach. WHO Regional Office for the Western Pacific. https://apps.who.int/iris/handle/10665/336862.

